# *Bartonella* spp. and *Rickettsia felis* in Fleas, Democratic Republic of Congo

**DOI:** 10.3201/eid1412.080610

**Published:** 2008-12

**Authors:** Cherilyn Sackal, Anne Laudisoit, Michael Kosoy, Robert Massung, Marina E. Eremeeva, Sandor E. Karpathy, Kristen Van Wyk, Elizabeth Gabitzsch, Nordin S. Zeidner

**Affiliations:** Centers for Disease Control and Prevention, Fort Collins, Colorado, USA (C. Sackal, M. Kosoy, K. Van Wyk, N. Zeidner); University of Antwerp, Antwerp, Belgium ( A. Laudisoit); University of Liege, Liege, Belgium (A. Laudisoit); Centers for Disease Control and Prevention, Atlanta, Georgia, USA (R. Massung, M.E. Eremeeva, S.E. Karpathy); Etubics Corporation, Seattle, Washington, USA (E. Gabitzsch)

**Keywords:** Bartonella, Rickettsia, flea-borne diseases, Democratic Republic of Congo, letter

**To the Editor:** *Bartonella* and *Rickettsia* species are pathogens of humans and domestic mammals that may be transmitted by fleas and other arthropods. *Rickettsia felis* causes flea-borne spotted fever in humans who come into contact with flea-infested domestic and peridomestic animals; worldwide distribution of this pathogen in ectoparasites and mammals makes it an emerging threat to human health (*1**,**2*). Likewise, species of the genus *Bartonella* are associated with an increasing array of human diseases, including trench fever, cat-scratch disease, and endocarditis in immunocompetent patients, and bacillary angiomatosis and peliosis hepatitis in immunocompromised patients (*3**–**5*). Although *Bartonella* spp. and *R.* *felis* appear to be globally distributed, their presence in the Democratic Republic of Congo (DRC) has not been previously documented.

Off-host *Pulex irritans*, *Tunga penetrans*, *Ctenocephalides felis strongylus*, *Echidnophaga gallinacea*, and *Xenopsylla brasiliensis* were collected in the Ituri district of northeastern DRC from March through April 2006, during an investigation of a plague outbreak. Our investigation area was limited to 4 villages: Djalusene and Kpandruma, which had confirmed plague patients, and Wanyale and Zaa, which had several suspect cases.

We collected fleas by using a kerosene lamp hung above a 45-cm diameter tray containing water (*7*). Captured fleas were identified using a dissecting microscope and standard morphologic keys, sorted into vials by species and locality, and preserved in 70% ethanol (*7*). Fleas were separated into 193 pools (2–5 fleas per pool), triturated for 10 minutes; the resultant flea triturate was centrifuged at 3,000 rpm for 10 minutes to collect flea tissue. DNA was then obtained by using the DNeasy Blood and Tissue Kit (QIAGEN, Valencia, CA, USA).

*Bartonella* DNA was detected by PCR amplifying a 379-bp fragment of the citrate synthase gene (*gltA*) (*8*). For *Rickettsia typhi* and *R. felis*, a real-time multiplex PCR assay targeting a conserved fragment of *glt*A was used (unpub. data). All assays were run in duplicate, and positive and negative controls were included in all assays. Amplicons were purified with the QIAquick PCR purification kit (QIAGEN) and sequenced in both directions by using a BigDye sequencing kit (Applied Biosystems, Foster City, CA, USA) with the same primers used for PCR amplification. Resultant sequences of *Bartonella* spp. were aligned with MegAlign by using the Clustal algorithm (DNASTAR, Inc., Madison, WI, USA), and compared with reference sequences obtained from GenBank.

Although *Yersinia* DNA and *R. typhi* were not detected, 89 of the 193 pools were PCR positive for either *Bartonella* spp. or *R. felis* (Table). Using the Microsoft Excel Add-In PooledInfRate software (Redmond, WA, USA; www.cdc.gov/ncidod/dvbid/westnile/software.htm), we calculated an estimated infection rate of 10.72% (95% confidence interval [CI] 8.52–13.31) for *R. felis*, 3.66% for *Bartonella* species, and 0.91% (95% CI 0.40–1.78) for both *Bartonella* spp. and R*. felis* (Table).

**Table:** Detection of *Bartonella* spp. and *Rickettsia* spp. DNA in fleas collected in the Democratic Republic of Congo*

Village	Flea species†	Identification no.	*Bartonella* spp.‡	*Rickettsia* spp.‡
Kpandruma	*Pulex irritans*	42d	*B. clarridgeiae*	
	*P. irritans*	44e	*B. vinsonii*	
	*P. irritans*	46	*B. vinsonii*	
	*P. irritans*	48a	*B. vinsonii*	
	***P. irritans***	**48b**	***B. vinsonii***	***R. felis***
	*P. irritans*	48e	Unique	
	*P. irritans*	49a	Unique	
	*P. irritans*	51b	*B. clarridgeiae*	
	*P. irritans*	41a		*R. felis*
	*P. irritans*	42b		*R. felis*
	*P. irritans*	43b		*R. felis*
	*P. irritans*	44a		*R. felis*
	*P. irritans*	94a		*R. felis*
	*P. irritans*	94b		*R. felis*
	*P. irritans*	94d		*R. felis*
	*P. irritans*	94e		*R. felis*
	*P. irritans*	94g		*R. felis*
	*P. irritans*	98		*R. felis*
	*P. irritans*	100a, 100b, 100d		*R. felis*
	*Xenopsylla brasiliensis*	102		*R. felis*
	*P. irritans*	113a, 113b		*R. felis*
	*Tunga penetrans*	114		*R. felis*
	*P. irritans*	123b		*R. felis*
Djalusene	*P. irritans*	22	*B. vinsonii*	
	*T. penetrans*	29	Identical to EU549693	
	*P. irritans*	61b	*B. clarridgeiae*	
	*P. irritans*	69f	Candidatus *B. rochalimae*	
	***Ctenocephalides felis strongylus***	**78**	***B. clarridgeiae***	***R. felis***
	*P. irritans*	84b	Candidatus *B. rochalimae*	
	***T. penetrans***	**85**	**Candidatus *B. rochalimae***	***R. felis***
	***P. irritans***	**86a**	**Candidatus *B. rochalimae***	***R. felis***
	*P. irritans*	86b-86d	Candidatus *B. rochalimae*	
	*T. penetrans*	33a		*R. felis*
	*P. irritans*	34d		*R. felis*
	*C. felis strongylus/P.irritans*	67		*R. felis*
	*P. irritans*	69d-69e		*R. felis*
	*T. penetrans/Echidnophaga gallinacea*	73		*R. felis*
	*E. gallinacea*	75c		*R. felis*
	*P. irritans*	80a-80b		*R. felis*
	*T. penetrans*	83		*R. felis*
	*P. irritans*	86c		*R. felis*
Wanyele	***P. irritans***	**52**	***B. vinsonii***	***R. felis***
	***C. felis strongylus***	**63a**	**Candidatus *B. rochalimae***	***R. felis***
	*E. gallinacea*	56		*R. felis*
	*C. felis strongylus*	63b		*R. felis*
	*C. felis strongylus*	88a,c,e		*R. felis*
	*T. penetrans*	103		*R. felis*
	*C. felis strongylus*	105a		*R. felis*
	*P. irritans*	106a		*R. felis*
	*T. penetrans*	107		*R. felis*
	*P. irritans*	106c		*R. felis*
	*P. irritans*	109		*R. felis*
	*P. irritans*	110		*R. felis*
	*C. felis strongylus*	111a		*R. felis*
Zaa	*P. irritans*	15	*B. vinsonii*	
	*P. irritans*	18	Candidatus *B. rochalimae*	
	*P. irritans*	95b	Candidatus *B. rochalimae*	
	***P. irritans***	**95c**	**Candidatus *B. rochalimae***	***R. felis***
	*C. felis strongylus*	66	*B. clarridgeiae*	
	*P. irritans*	6c, 6f		*R. felis*
	*E. gallinacea*	12b		*R. felis*
	*P. irritans*	17		*R. felis*
	*P. irritans*	20a-20c		*R. felis*
	*P. irritans*	24		*R. felis*
	*P. irritans*	39b		*R. felis*
	*C. felis strongylus/P. irritans*	64a		*R. felis*
	*P. irritans*	64b		*R. felis*
	*P. irritans*	93b-93d		*R. felis*
	*P. irritans*	95g,h,l,m		*R. felis*
	*P. irritans*	115b		*R. felis*
	*P. irritans*	117		*R. felis*
	*P. irritans*	119		*R. felis*

*Boldface represents dual infection. †Identified using standard taxonomic keys. ‡Detected by PCR as described in the methods.

Phylogenetic analysis indicated several *Bartonella* spp. in fleas that were closely aligned with pathogenic *Bartonella* spp., including *B. vinsonii*, *Candidatus* *B. rochalimaea*, and *B. clarridgeiae* (data not shown). Moreover, *Bartonella* from 3 pools of *P. irritans* demonstrated only 1.8% to 2.4% divergence to *B. vinsonii* subspecies *arupensis* isolated from a human patient in Wyoming, USA. Likewise, sequences of *Bartonella* from 1 pool of *T. penetrans* and 1 pool of *P. irritans* were 100% identical to *Bartonella* isolated from a *Neotoma mexicana* wood rat (GenBank accession no. AF110312); a sequence obtained from 1 flea pool of *T. penetrans* was 100% identical to the *glt*A *Bartonella* sequence found in *Orchopeas sexdentatus,* collected from *Neotoma micropus* in New Mexico, USA (data not shown). This finding indicates a new *Bartonella* species with multiple rodent origins and a more ubiquitous global dissemination than previously determined. Our results also demonstrate the previously unreported detection of *R. felis* in *P. irritans*, *E. gallinacea*, *X. brasiliensis*, and *T. penetrans* flea species.

This report suggests that *Bartonella* spp. and *R. felis* exists in fleas within the DRC. In addition, we report *Bartonella* spp. and *R. felis* DNA in *T. penetrans* fleas and *R. felis* DNA in *E. gallinacea* fleas, vectors not previously associated with these pathogens. Co-infections were also observed in *T. penetrans*, *P. irritans*, and *C. felis* fleas, suggesting a common vector or mammalian host shared by *R. felis* and *Bartonella* spp. Flea feedings occur intermittently and on potentially different hosts, thus the vectors described here may acquire multiple bacterial strains for transmission to humans (*8*). Moreover, PCR assays targeting the cytochrome B gene indicated human blood in the flea pools, demonstrating dual infection (data not shown); this finding shows that the flea species recovered are capable of feeding on humans, have a broad host range, and are capable of transmitting disease to humans (*9*).

*Bartonella* spp. and *R. felis* have been detected previously in fleas within northern and sub-Saharan Africa (*10*). The presence of *Bartonella* spp. and *R. felis* in the fleas is important because they were collected in close contact with humans at risk for multiple exposures within households. Our results suggest that both *R. felis* and *Bartonella* spp. are prevalent in this region of the DRC and should be included in the differential diagnosis of potential flea-borne infections in this region of sub-Saharan Africa**.**
